# Systematic review with network meta-analysis: statins and risk of hepatocellular carcinoma

**DOI:** 10.18632/oncotarget.7832

**Published:** 2016-03-01

**Authors:** Yao-Yao Zhou, Gui-Qi Zhu, Yue Wang, Ji-Na Zheng, Lu-Yi Ruan, Zhang Cheng, Bin Hu, Shen-Wen Fu, Ming-Hua Zheng

**Affiliations:** ^1^ Department of Cardiology, Jinhua Municipal Hospital, Jinhua 321004, China; ^2^ Department of Infection and Liver Diseases, Liver Research Center, The First Affiliated Hospital of Wenzhou Medical University, Wenzhou 325000, China; ^3^ School of The First Clinical Medical Sciences, Wenzhou Medical University, Wenzhou 325000, China; ^4^ Institute of Hepatology, Wenzhou Medical University, Wenzhou 325000, China

**Keywords:** statins, hepatocellular carcinoma, network meta-analysis, indirect comparison

## Abstract

**Objectives:**

Usage of statins is suggested to decrease the incidence of HCC. When it comes to different statin subtypes, the chemopreventive action remains controversial. We aim to compare the usage of different statins and reduction of HCC risk.

**Methods:**

We searched PubMed, Embase.com and Cochrane Library database up to August 10, 2015. Duplicated or overlapping reports were eliminated. We performed a traditional pair-wise meta-analysis and a Bayesian network meta-analysis to compare different treatments with a random-effects model.

**Results:**

We reviewed five observational studies enrolling a total of 87127 patients who received at least two different treatment strategies including rosuvastatin, atorvastatin, simvastatin, pravastatin, fluvastatin, cerivastatin, and lovastatin or observation alone. Direct comparisons showed that usage of atorvastatin (OR 0.63, 95%CI 0.45-0.89) and fluvastatin (OR 0.58, 95%CI 0.40-0.85) could significantly cut the risk of liver cancer. The difference of indirect comparisons between the included regimens is not statistically significant. However, usage of all types of statins, such as fluvastatin (RR 0.55, 95%CI 0.26-1.11), atorvastatin (RR 0.59, 95%CI 0.30-1.16), simvastatin (RR 0.69, 95%CI 0.38-1.25), cerivastatin (RR 0.71, 95%CI 0.19-2.70), pravastatin (RR 0.72, 95%CI 0.37-1.45), lovastatin (RR 0.81, 95%CI 0.34-1.96) and rosuvastatin (RR 0.92, 95%CI 0.44-1.80), appeared to be superior to observation alone. Notably, fluvastatin was hierarchically the best when compared with the six other statins.

**Conclusions:**

Our analyses indicate the superiority of usage of statins in reduction of liver cancer. Available evidence supports that fluvastatin is the most effective strategy for reducing HCC risk compared with other statin interventions.

## INTRODUCTION

Hepatocellular carcinoma (HCC) is recognized as the sixth most prevalent malignancy and annually accounts for one of the most common causes of cancer deaths worldwide. HCC has a dismal 5-year survival rate, and to date there is no effective chemotherapy treatment [1, 2].

Statins are inhibitors of 3-hydroxy-3-methylglutaryl coenzyme A (HMG-CoA) reductase, which is a key enzyme in the rate-limiting step in cholesterol synthesis. Statins are used as potent cholesterol-lowering medications in the primary and secondary prevention of cardiovascular and cerebrovascular disease [3]. Contrary to previous concerns over the carcinogenicity of statins [4], a growing body of studies suggest statins may have an unexpected benefit for reducing cancer [5]. Evidence from *in vitro* and *in vivo* experimental studies have shown that statins have potential protective effects against primary and metastatic HCC via induction of apoptosis, arrest of cell proliferation, and inhibition of angiogenesis [6].

Results of human studies, however, remain controversial as to a beneficial role of statins in either preventing or curing patients presenting with HCC. Two randomized controlled trials showed that additional use of pravastatin prolonged the survival of patients with advanced HCC [7, 8]. Observational studies based on a computerized population database also reported an inverse association between use of statins and the incidence of HCC [9]. However, results from these cohort studies need to be interpreted with caution on account of methodological limitations such as selection bias or confounders. Promising evidence that statins may decrease the risk of HCC has been generated from recent meta-analyses, suggesting statins as a potential adjuvant therapy in the treatment of liver cancer [10–12]. However, the Cholesterol Treatment Trialists' Collaboration study, using individual patient records from 175,000 randomized patients, has failed to show any decrease in incidence or mortality for any type of cancer, including HCC [13].

To date, there are no large randomized controlled trials assessing the potential effects of statin therapies in patients either susceptible to develop HCC or with advanced disease. Given the ambiguous results and limited epidemiological evidence for a link between statin use and risk of liver cancer, the current study sought to evaluate the association of statin use and the risk of HCC. Furthermore, we sought to define potential chemoprevention strategies for the treatment of HCC for respective statin subtypes. To attain a better understanding on this issue, we performed an updated Bayesian network-analysis of available observation studies to investigate the association between use of different types of statins and the risk of developing HCC.

## RESULTS

### Study characteristics

**Figure 1:**
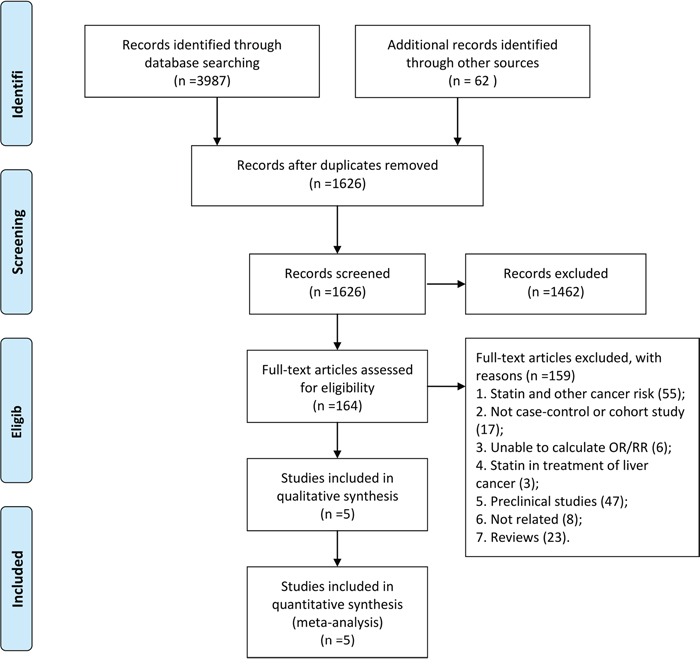
Study selection

**Figure 2:**
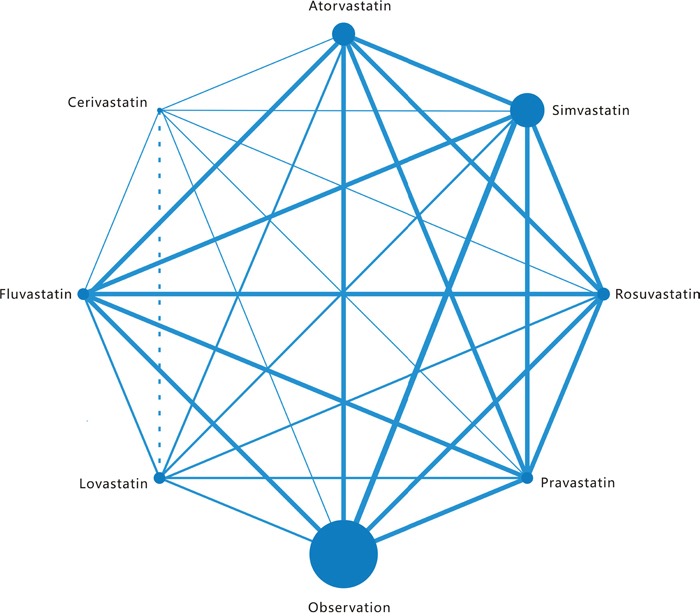
Network of the comparisons for the Bayesian network meta-analysis The size of every node is proportional to the number of patients. Lines connect the interventions that have been studied in head- to-head (direct) comparisons in the eligible studies. The width of the lines is proportional to the number of studies. For each intervention of interest, two or more eligible trials are involved in the respective networks.

The PRISMA flowchart showing the electronic searching processes is shown in Figure 1. The combined electronic and reference searches recovered 4049 potential relevant articles and after the initial screening, 2423 publications were excluded according to title and abstract. After detailed assessment of the full text, a further 1462 were excluded because they were not case control or cohort studies, without available data with respect to liver cancer, or were animal or basic research studies or review articles. Overall, five observational studies from different countries enrolling a total of 87127 patients who received at least two different treatment strategies were included in this analysis (Figure 2) [14–18]. The details of the interventions, baseline characteristics of the populations, study period, and adjusted confounders of five eligible trials were outlined for network meta-analysis in Table 1. We included a total of eight regimens according to these eligible studies: rosuvastatin, atorvastatin, simvastatin, pravastatin, fluvastatin, cerivastatin, and lovastatin or observation alone. In terms of study sample sizes, the number of subjects involved in the studies ranged from 5835 to 33413. Among the five studies, which were most multiple-arm trials, patients were treated with simvastatin or observation alone in all studies, rosuvastatin, atorvastatin, pravastatin, and fluvastatin in five studies, lovastatin in two studies, and cerivastatin in only one study. Table 2 summarizes the quality assessment and scores of included studies, which showed that the quality of included studies were reliable.

**Table 1:** Characteristics of included studies

Studies	Design	Location	Patient population	Study period	Cases defined	Total no. of subjects	No. of HCC cases	Adjusted confounders^a^	Statin types^b^
McGlynn et al^15^, 2015	Case-control	UK	General population (CPRD)	1988-2011	Read codes B150300, B150z00, B152.00	5835	1195	5, 6, 8, 9, 11, 13, 14, 16, 20	R,A,S,P,F,C
Bergman et al^16^, 2014	Case-control	Sweden	General population (SPDR)	2006-2010	ICD-0/3 C22	23964	3994	4, 8-14, 20	R,A,S,P,F
Lai et al^17^, 2013	Case-control	Taiwan	General population (NHIRD)	2000–2009	ICD-9-CM 155	17400	3480	1, 2, 8-13, 15-18	R,A,S,P,F,L
El-Serag et al^18^, 2009	Case-control	USA	Diabetes patients (VA)	2001–2002	ICD-9-CM 155	6515	1303	1-3, 7-11, 13, 14, 16-19	S
Tsan et al, 2012^19^	Cohort	Taiwan	Patients with HBV infection (NHIRD)	1997–2008	ICD-9 155	33, 413	1021	1, 2, 4, 10, 13, 14	R,A,S,P,F,L

a 1, age; 2, sex; 3, race; 4, socioeconomic status; 5, body mass index; 6, smoking; 7, ethanol intake; 8, HBV infection; 9, HCV infection; 10, cirrhosis; 11, alcoholic liver disease; 12, on-alcoholic liver disease; 13, diabetes mellitus; 14, cardiovascular medications (aspirin/nonsteroidal anti-inflammatory medications, angiotensin-converting enzymes inhibitors); 15, other lipid-lowering agents; 16, antidiabetics; 17, treatments for HBV; 18, treatments for HCV; 19, propensity to use statins; 20, medications taken (unspecified).

b R: Rosuvastatin, A: Atorvastatin, S: Simvastatin, P: Pravastatin, F: Fluvastatin, C: Cerivastatin, L: Lovastatin.

**Table 2:** Quality assessment of included studies

Authors	Year	Selection				Comparability	Outcome or exposure			Score
		1	2	3	4	5	6	7	8	
McGlynn et al^15^	2015		*	*	*	**	*	*	*	********
Bergman et al^16^	2014		*	*	*	**	*	*	*	********
Lai et al^17^	2013		*	*	*	**	*	*	*	********
El-Serag et al^18^	2009		*	*	*	**	*	*	*	********
Tsan et al^19^	2012		*	*	*	**	*	*	*	********

### Results from pair-wise comparisons

**Figure 3:**
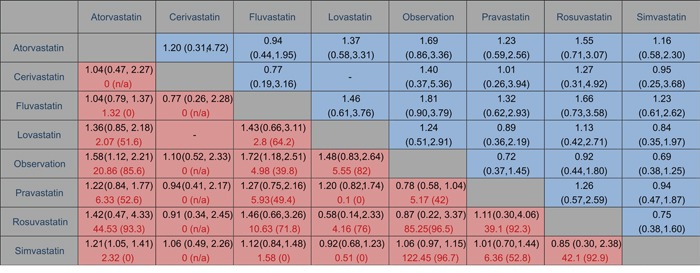
Comparison of outcomes of different statin treatment between pair-wise meta-analysis and network meta-analysis The cells in red are results of pair-wise meta-analysis in the first line, with assessment of heterogeneity as I^2^ (%) in second line marked in red. The row treatment is compared with the column treatment. On the other side, results of network meta-analysis are in blue cells. The column treatment is compared with the row treatment. Ranges in parentheses are 95% CIs.

Pairwise meta-analysis was accomplished for the eight different comparisons, with a lack of direct comparison between cerivastatin and lovastatin. The weighted RRs for the HCC occurrence were calculated for each comparison, the geometric distribution of which was displayed in Figure 3. Specifically, meta-analysis of the direct comparisons showed that usage of atorvastatin (OR 0.63, 95% CI 0.45- 0.89) and fluvastatin (OR 0.58, 95% CI 0.40- 0.85) could cut the risk of liver cancer, while other types of statin failed to suggest a borderline significant efficacy compared with observation alone. Additionally, in comparisons between active interventions, atorvastatin appeared to be superior to simvastatin (RR 0.83, 95% CI 0.71- 0.95) significantly, together with other subtypes such as fluvastatin (RR 0.96, 95% CI 0.73–1.27), cerivastatin (RR 0.96, 95% CI 0.44–2.13), lovastatin (RR 0.74, 95% CI 0.46–1.18), pravastatin (RR 0.82, 95% CI 0.56–1.19), and rosuvastatin (RR 0.70, 95% CI 0.23–2.13), although differing without significance. These results arose from 27 independent analyses.

With respect to statistical heterogeneity, it was assessed in two of the comparisons by the I^2^ statistic. Overall, statistical heterogeneity was moderate, although for some comparisons 95% CIs were wide and included values indicating very high or no heterogeneity. In the meta-analyses of direct comparisons for efficacy, I^2^ values higher than 75% were recorded for seven comparisons. In addition, all P values for Begg's rank correlation test and Egger's test were difficult to assess due to the limited amount of research.

### Results from the network meta-analysis

**Figure 4:**
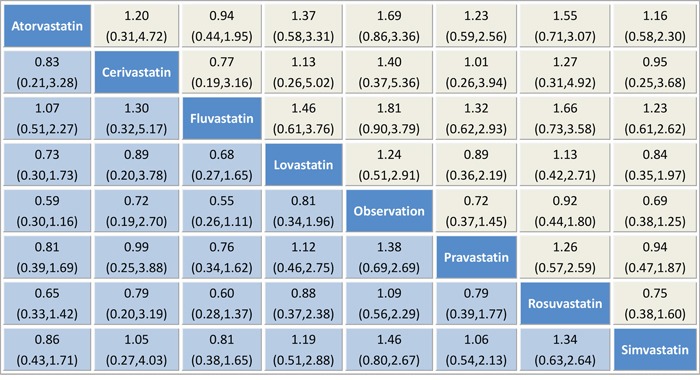
Pooled odds ratios for HCC recurrence The column treatment is compared with the row treatment. Accordingly, “treatment A vs treatment B” is not the same as “treatment B vs treatment A”, which is in the yellow and blue squares respectively. Numbers in parentheses indicate 95% credible intervals.

We summarized the results of the random-effects network meta-analysis for HCC rates in Figure 4, which illustrated the ORs for HCC rate with 95% confidence intervals obtained from the indirect comparisons of the included regimens. Generally, the difference of indirect comparisons between the included regimens was not statistically significant. Still, usage of all types of statins, such as fluvastatin (RR 0.55, 95% CI 0.26–1.11), atorvastatin (RR 0.59, 95% CI 0.30–1.16), simvastatin (RR 0.69, 95% CI 0.38–1.25), cerivastatin (RR 0.71, 95% CI 0.19–2.70), pravastatin (RR 0.72, 95% CI 0.37–1.45), lovastatin (RR 0.81, 95% CI 0.34–1.96) and rosuvastatin (RR 0.92, 95% CI 0.44–1.80), appeared to be superior to observation alone. Notably, fluvastatin showed a trend to more beneficial effects when compared with the six other statins, namely, atorvastatin (RR 0.94, 95% CI 0.44–1.95), simvastatin (RR 0.81, 95% CI 0.38–1.64), cerivastatin (RR 0.77, 95% CI 0.19–3.16), pravastatin (RR 0.76, 95% CI 0.34–1.61), lovastatin (RR 0.68, 95% CI 0.27–1.64) and rosuvastatin (RR 0.60, 95% CI 0.28–1.37), albeit with no statistical significance achieved.

**Figure 5:**
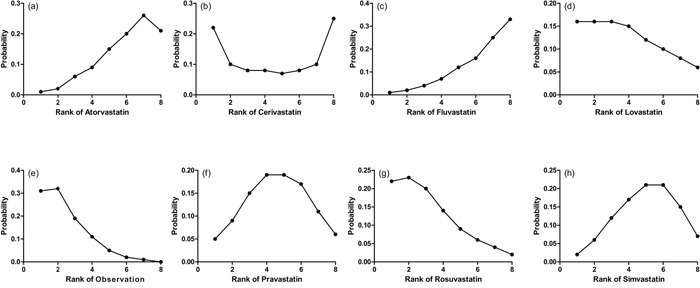
Ranking for recurrence of each intervention for HCC Ranking indicates the probability to be the best treatment, the second best, the third best and so on. Rank 1 is worst and rank N is best.

**Figure 6:**
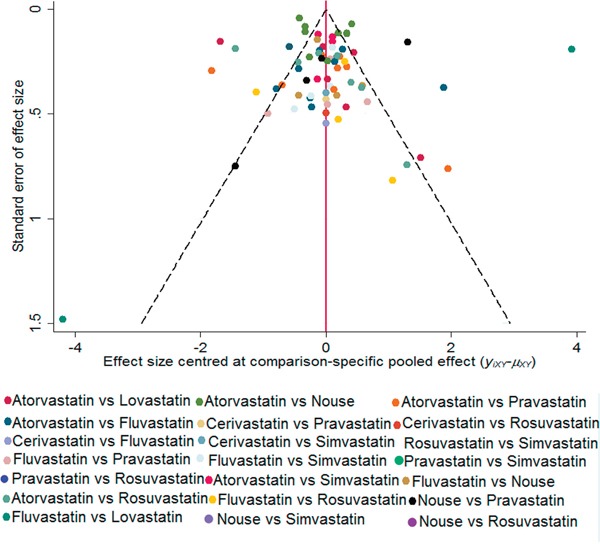
Comparison-adjusted funnel plot The red dotted line represents the null hypothesis that the study-specific effect sizes cannot differ from the respective comparison-specific pooled effect estimates. The two black dashed lines represent a 95% CI for the difference between study-specific effect sizes and comparison-specific summary estimates. Different colors correspond to different comparisons. yixy is the noted effect size in study i that compares x with y. μxy is the comparison-specific summary estimate for x versus y.

The probabilities of best treatment for each intervention were ranked at each of the eight possible parameters (Figure 5). Consistently, fluvastatin was hierarchically judged the best to cut the risk of HCC according to the estimated surface under the cumulative curve values, suggesting that fluvastatin was more efficacious than other remaining interventions. Otherwise, irrespective of observation alone, rosuvastatin was ranked the lowest in the prevention of HCC, which may suggest that it was least effective in reducing HCC rate. Figure 6 was an extension of the common funnel plot in cases of multiple treatment comparisons, without evidence of asymmetry, suggesting the absence of any small study effects or publication bias.

### Comparisons between traditional pairwise and network meta-analyses

Figure 3 showed the results of traditional pairwise and network meta-analyses. Although the pooled estimates showed small differences, the CIs from traditional pairwise meta-analyses and the Bayesian network meta-analyses in general overlapped, suggesting the evidence derived from both methods are consistent.

## DISCUSSION

This present network meta-analysis, which was based on five observational studies involving 87127 people and 10993 liver cancer cases, was designed to investigate the association between different types of statin use and the risk of liver cancer. Our results showed that compared with observation alone, seven different types of statin conferred relative benefits to decrease HCC incidence. As presented in this network meta-analysis, fluvastatin specifically appeared to provide an overall advantage on reduction in HCC risk over the remaining interventions and was ranked first among the seven types of statins. Moreover, the likelihood of important selection or publication bias was small.

Statins have been routinely used in patients with cardiovascular diseases thanks to their notable lipid-lowering effects. Despite the potential benefits of statin use in reduction of HCC, concerns about widespread adoption of this strategy are justified, because information on the balance between potential benefits and safety are scarce. Although there were concerns over carcinogenicity of statins raised by early animal studies, recent evidence has supported a potential chemopreventive role against liver cancer [19, 20], although, there remains no consensus reached from the different preclinical or epidemiological studies. Overall, we found that statin use was associated with risk reduction in liver cancer in different degree compared with observation, regardless of the type of statin. This result was in line with the previous meta-analyses, which suggested a favorable effect of statins on HCC in the same manner. Three recent traditional met-analyses found a summary RR of 0.58 (95%CIs 0.46 to 0.74), 0.63 (95%CIs 0.52 to 0.76) and 0.61 (95%CIs 0.49 to 0.76), respectively, which similarly suggested a favorable effect of statins on HCC [11, 12]. Shi et al. further concluded that the preventive effect of statin on liver cancer when taken daily for cardiovascular event prevention might be overestimated on account of residual confounders such as the exposure period, the indication and contraindication of statins [10]. Thus, more high quality prospective intervention trials are warranted to clarify this relationship.

Based on the evidence that the growth of HCC is critically dependent upon cholesterol, as well as statin selective localization to the liver and less than 5% reaches the circulatory system, statins are proposed to have a specific role in liver carcinogenesis. It is plausible that lipophilic statins (eg, lovastatin, simvastatin, atorvastatin, fluvastatin) may be superior to hydrophilic statins (eg, pravastatin, rosuvastatin) in liver cancer preventive qualities owing to better lipid solubility and membrane permeability [21, 22]. Conversely, there were no significant differences in the risk reduction of liver cancer with hydrophilic or lipophilic statins reported in several studies, which is also in line with our findings [10, 12, 18]. The molecular basis accounting for statins affecting the pathogenesis and biological features of HCC has been explored from different aspects. Firstly, statins regulate critical cellular functions related to cancer growth and metastasis such as maintenance of membrane integrity, signal transduction, protein synthesis, and cell proliferation by inhibiting HMG-CoA reductase, the rate-limiting enzyme in the mevalonate and lipogenic pathway [19]. Secondly, activation of the proto-oncogene Myc is recognized as a key step in liver oncogenesis. Statins are potent in treatment of MYC-associated HCC by inhibiting HMG-CoA reductase, which facilitates MYC phosphorylation, activation, and hepatocarcinogenesis specifically [23]. Thirdly, by inhibiting prenylation of small GTPases, which serve as crucial molecular switch that controls various signal transduction pathways, statins are also able to mediate cell migration and metastasis [5].

There are several strengths to consider in our analysis. It is worth mentioning that our current study is the first Bayesian network meta-analysis performed on the relationship of subtypes of statin use and the reduced risk of HCC. Apart from direct active comparisons, our network meta-analysis figured out a comprehensive and complete picture to get precise effect estimates of respective type of statin, even when no head-to-head studies existed. First, this network meta-analysis compares all available statin therapies simultaneously and assesses each therapy individually, rather than only grouping them into statin use or not. Secondly, a rankogram of available types of stain for chemoprevention of HCC provides a formal rank order for treatment strategies. Application of a Bayesian network meta-analysis contributes to a general evaluation of multiple statin strategies [24, 25]. In addition, statistical heterogeneity was moderate, although for some I^2^ values of comparisons, heterogeneity was high.

Our network meta-analysis has to be interpreted with caution in view of some limitations. First, most studies included in this analysis are observational but not prospective studies, which might affect the validity of overall findings. To our knowledge, by reason of the low incidence of liver cancer, it is difficult to complete a large randomized controlled trial within a finite time horizon. Secondly, owing to scant primary data, the size of each subtype group is particularly small after divided into different types of statins. The present debate about optimum statin treatments to prevent HCC will be assisted greatly by collection of robust data in future trials. Thirdly, the characteristics of populations were different, which mainly come from Asian, Europe, and North America, and yet few data was available from other countries or races. Fourth, more detailed data for duration or dosage of statin therapies is barely available, which may make a difference to the outcomes. Besides, there are some other confounders including different causes of HCC such as HBV/HCV infections or alcoholic liver disease, concomitant diseases, anti-viral therapies of HBV or HCV and so on, which might overestimate the preventive effect of statins as mentioned above. Lastly, statistical heterogeneity was moderate in the meta-analyses of direct comparisons, while no substantial inconsistency was found in the network meta-analysis.

In summary, this network meta-analysis provides a useful and complete picture which convinced the associations between statin use and reduced HCC risk by using Bayesian analytical approach. Our analysis shows the superiority of usage of statins in reduction of liver cancer. Among all, fluvastatin is the most effective strategy for reducing HCC risk compared with other statin interventions. Our analysis of up-to-date evidence may provide new insights into controversies on this issue with valuable implications in clinical care and future research. Still, there is a strong need for well-designed randomized controlled trials for the chemoprevention of HCC.

## MATERIALS AND METHODS

### Search strategy

The protocol for the systematic review was performed in accordance with PRISMA (Preferred Reporting Items for Systematic Reviews and Meta-Analyses) guideline [26]. We searched PubMed, Embase and the Cochrane Library databases with the key terms ‘statin, hepatocellular carcinoma, and humans’ without language or date restrictions up to 31 August 2015. We manually searched bibliographies of published articles and literature searches were complemented by perusing the reference lists of previous meta-analyses.

### Selection criteria

In our trials for meta-analysis, included trials had to meet the following criteria: (1) RCTs, cohort studies or case-control studies; (2) original studies designed to assess the association between the use of statin and the risk of liver cancer with specific classification; (3) interventions: treatment with all kinds of statin treatments or observation alone; (4) population: no prior diagnosis of primary liver cancer or other cancers most likely to metastasize to the liver, such as stomach, colon, breast and so on; (5) risk estimates should be provided or could be calculated with sufficient usable outcomes data. Duplicated or overlap reports were eliminated according to the same title, author list or publication date. If more than one paper was derived from the same population, otherwise, only data from the most recent published report was included. Eligible studies should be published in the form of peer review articles in full length without language restrictions. Eventually, a total of five observational studies were included, which were all published in English though.

### Data extraction

Two investigators (Zhou YY, Zhu GQ) independently reviewed the full manuscripts of eligible studies and extracted information into an electronic database: patients' characteristics study design, interventions, type of statins, adjusted confounders, the number of events of interest in each group. Any discrepancies regarding the extraction of data were resolved by an additional investigator (Zheng MH). When relevant information on design or outcomes was unclear, or when some needed data was unavailable directly from the study, the original authors were sought for eligible data by email.

### Quality assessment

The quality of the methodology was independently assessed by two reviewers using the Newcastle-Ottawa Quality Assessment Scale with some modifications to match the needs of this study. The quality of the studies was evaluated by a ‘star system’ based on three items: patient selection, comparability of statin and observation group, and the ascertainment of either the exposure or outcome of interest for case-control or cohort studies respectively [27].

### Data analysis

First, we performed a traditional pair-wise meta-analysis which could synthesize studies that compared the same interventions directly by using STATA 12.0 (Stata Corporation, College Station, Texas, USA). Then a Bayesian network meta-analysis to compare different treatments (rosuvastatin, atorvastatin, simvastatin, pravastatin, fluvastatin, cerivastatin, and lovastatin, as well as observation) to each other was followed respectively, with a random-effects model using Markov chain Monte Carlo methods in WinBUGS (MRC Bio-statistics Unit, Cambridge, UK) as described in our previous work [28–30]. The DerSimonian and Laird random effects model was utilized to calculate pooled estimates of hazard ratios (HRs), relative risks (RRs), and 95% confidence intervals (CIs) of direct comparisons between two strategies according to Cochrane Handbook for Systematic Reviews of Interventions Version 5.1.0. Clinical heterogeneity was first assessed through clinical judgment with input from experts in the field. A formal confirmation of heterogeneity was then obtained by referring to the I^2^ statistic, which if >50% suggested statistically significant heterogeneity, if between 25% and 50% considered moderate heterogeneity levels, and if <25% implied low heterogeneity levels.

We also estimated the relative ranking probability that which treatment was the most efficacious therapy, the second best, the third best and so on. Treatment regimens were ranked in terms of HCC occurrence with the same methodology. Taken together, the multiple-treatments meta-analysis raised statistical power by incorporating evidence from both direct and indirect comparisons across all interventions.

## Abbreviations

CIs: confidence intervals
HCC: hepatocellular carcinoma
HMG-CoA: 3-hydroxy-3-methylglutaryl coenzyme A
HRs: hazard ratios
PRISMA: Preferred Reporting Items for Systematic Reviews and Meta-Analyses
RRs: relative risks
